# Supplementary material for: Candidate Regulatory Genes for Hindlimb Development in the Embryos of the Chinese Alligator (Alligator sinensis)
Source: Animals (Basel). 2023 Oct 6;13(19):3126. doi: 10.3390/ani13193126 (PMC10571561; doi:10.3390/ani13193126)
Supplement: Supplementary file 1 [file animals-13-03126-s001.zip › animals-2550268-supplementary.pdf]

Table S1. Real-time quantitative PCR primers used in this study.

Locus	primer sequence（5'→3'）	Annealing temperature	Amplified fragment length
β-actin	F:ACCGAAACAAGAACCCAT<br>R:CCGACACGATAAGACTGC	56	133
CAV1	F:TGCCGAACCAGAGGGAACAC<br>R:GCCCAGATGTGCAGGAATGAC	56	171
IRS2	F:CAGTGGTGGCGCAGAACAT<br>R:GGTAATGGGGTGCGTAGCTG	56	155
LDB3	F:TCGACTTCAGCCTACCCAAC<br>R:TACCAGGAAAGGACCCCGAA	56	153
LDHA	F:TGTCCACAAGGAAGAGCACG<br>R:CTCTCAGCTTGTCCTCCACG	56	149
MYL3	F:TGAAACCTTCCTGCCGATGCT<br>R:ACCTCCTCTTCAGTCAGCCTCT	56	174

Table S2. Genome alignment analysis

Samples	total_reads	total_map	unique_map	multi_map	positive_map	negative_map
A1	42100124	38410013<br>(91.23%)	37326929<br>(88.66%)	1083084<br>(2.57%)	18665393<br>(44.34%)	18661536<br>(44.33%)
A2	40232798	36794914<br>(91.46%)	35958629<br>(89.38%)	836285<br>(2.08%)	17978015<br>(44.68%)	17980614<br>(44.69%)
A3	39816432	36314397<br>(91.2%)	35410874<br>(88.94%)	903523<br>(2.27%)	17708823<br>(44.48%)	17702051<br>(44.46%)
A4	40533992	37099191	36229269	869922	18115567	18113702

		(91.53%)	(89.38%)	(2.15%)	(44.69%)	(44.69%)
B1	43250912	39658978 (91.7%)	38751589 (89.6%)	907389 (2.1%)	19380485 (44.81%)	19371104 (44.79%)
B2	39288900	35745398 (90.98%)	34724163 (88.38%)	1021235 (2.6%)	17375746 (44.23%)	17348417 (44.16%)
B3	44464890	40724353 (91.59%)	39786913 (89.48%)	937440 (2.11%)	19900966 (44.76%)	19885947 (44.72%)
B4	44434414	40601311 (91.37%)	39580767 (89.08%)	1020544 (2.3%)	19796629 (44.55%)	19784138 (44.52%)
C1	44081482	40220568 (91.24%)	39015390 (88.51%)	1205178 (2.73%)	19524369 (44.29%)	19491021 (44.22%)
C2	44523064	40339249 (90.6%)	38980266 (87.55%)	1358983 (3.05%)	19494937 (43.79%)	19485329 (43.76%)
C3	44572368	40596027 (91.08%)	39430578 (88.46%)	1165449 (2.61%)	19716378 (44.23%)	19714200 (44.23%)
C4	44076128	40021520 (90.8%)	38718860 (87.85%)	1302660 (2.96%)	19375700 (43.96%)	19343160 (43.89%)
D1	44062570	40019621 (90.82%)	38723817 (87.88%)	1295804 (2.94%)	19379380 (43.98%)	19344437 (43.9%)
D2	43390542	39315419 (90.61%)	38055392 (87.7%)	1260027 (2.9%)	19031787 (43.86%)	19023605 (43.84%)
D3	44144060	40195997 (91.06%)	38848201 (88.0%)	1347796 (3.05%)	19436840 (44.03%)	19411361 (43.97%)
D4	45648164	41429101 (90.76%)	40142912 (87.94%)	1286189 (2.82%)	20092699 (44.02%)	20050213 (43.92%)

total_reads：The number of clean reads of sequencing data after quality control

total_map：**Compare the number and percentage of reads on the genome**
unique_map: **Compare the number and percentage of reads to the unique location of the reference genome**
multi_map：**The number and percentage of reads to multiple locations of the reference genome were compared**
positive_map：**Compare the number and percentage of reads on the positive chain of the reference genome**
negative_map：**Compare the number and percentage of reads on the negative strand of the reference genome**

Table S3. The significantly enriched GO molecular function and cellular component terms

	modules	GO ID	Description	pvalue
Cellular Component	Blue	GO:0043292	contractile fiber	0.000000
		GO:0016529	sarcoplasmic reticulum	0.000000
		GO:0042383	sarcolemma	0.000000
		GO:0032982	myosin filament	0.000097
		GO:1902495	transmembrane transporter complex	0.000754
		GO:0031143	pseudopodium	0.000772
		GO:1990351	transporter complex	0.001308
		GO:1904813	ficolin-1-rich granule lumen	0.009059
		GO:0062023	collagen-containing extracellular matrix	0.010735
	Green	GO:0043209	myelin sheath	0.000000
		GO:0097386	glial cell projection	0.000015
		GO:0016323	basolateral plasma membrane	0.000209
		GO:0005922	connexin complex	0.000326
		GO:0098574	cytoplasmic side of lysosomal membrane	0.017790
		GO:1990531	phospholipid-translocating ATPase complex	0.017790
		GO:0031092	platelet alpha granule membrane	0.021562
		GO:0043025	neuronal cell body	0.025296

		GO:0008305	integrin complexBlue	0.038983
		GO:0098636	protein complex involved in cell adhesion	0.070549
	Grey	GO:0030672	synaptic vesicle membrane	0.000313
		GO:0099501	exocytic vesicle membrane	0.000313
		GO:0048786	presynaptic active zone	0.003477
		GO:0071546	pi-body	0.010710
		GO:0033391	chromatoid body	0.012839
		GO:0043194	axon initial segment	0.022364
		GO:0008076	voltage-gated potassium channel complex	0.080692
	Turquoise	GO:0062023	collagen-containing extracellular matrix	0.000000
		GO:0097060	synaptic membrane	0.000000
		GO:0098982	GABA-ergic synapse	0.000000
		GO:0005871	kinesin complex	0.000005
		GO:0071162	CMG complex	0.000006
		GO:0043198	dendritic shaft	0.000008
		GO:0016342	catenin complex	0.000018
		GO:0005581	collagen trimer	0.000118
		GO:0000307	cyclin-dependent protein kinase holoenzyme complex	0.000425
		GO:0034702	ion channel complex	0.000708
		GO:0043025	neuronal cell body	0.000784
		GO:0030496	midbody	0.001611
		GO:1902711	GABA-A receptor complex	0.014671
		GO:0030672	synaptic vesicle membrane	0.022955

		GO:0099501	exocytic vesicle membrane	0.022955
		GO:0005912	adherens junction	0.033473
		GO:0030118	clathrin coat	0.040547
		GO:0042627	chylomicron	0.041973
		GO:0042622	photoreceptor outer segment membrane	0.071001
		GO:0005903	brush border	0.071336
molecular function	Blue	GO:0043292	contractile fiber	0.000000
		GO:0016529	sarcoplasmic reticulum	0.000000
		GO:0042383	sarcolemma	0.000000
		GO:0032982	myosin filament	0.000097
		GO:1902495	transmembrane transporter complex	0.000754
		GO:0031143	pseudopodium	0.000772
		GO:1990351	transporter complex	0.001308
		GO:1904813	ficolin-1-rich granule lumen	0.009059
		GO:0062023	collagen-containing extracellular matrix	0.010735
	Green	GO:0019911	structural constituent of myelin sheath	0.000097
		GO:0005243	gap junction channel activity	0.000116
		GO:0005506	iron ion binding	0.001118
		GO:0098632	cell-cell adhesion mediator activity	0.002177
		GO:0043208	glycosphingolipid binding	0.013530
		GO:0016725	oxidoreductase activity, acting on CH or CH2 groups	0.014873
		GO:0140333	glycerophospholipid flippase activity	0.017555
		GO:0140327	flippase activity	0.021564
		GO:0008525	phosphatidylcholine transporter activity	0.022896

		GO:0046624	sphingolipid transporter activity	0.022896
		GO:0097001	ceramide binding	0.025557
		GO:0005452	solute:inorganic anion antiporter activity	0.026884
		GO:0043027	cysteine-type endopeptidase inhibitor activity involved in apoptotic process	0.028210
		GO:0033691	sialic acid binding	0.029534
		GO:0004435	phosphatidylinositol phospholipase C activity	0.032177
		GO:0051537	2 iron, 2 sulfur cluster binding	0.033496
		GO:0070840	dynein complex binding	0.033496
		GO:0004629	phospholipase C activity	0.036129
		GO:0140326	ATPase-coupled intramembrane lipid transporter activity	0.036129
		GO:0035250	UDP-galactosyltransferase activity	0.037443
		GO:0046625	sphingolipid binding	0.037443
		GO:0071949	FAD binding	0.051781
		GO:0004860	protein kinase inhibitor activity	0.053074
		GO:0043014	alpha-tubulin binding	0.055656
		GO:0048156	tau protein binding	0.056944
		GO:0019210	kinase inhibitor activity	0.058230
		GO:0019894	kinesin binding	0.059515
		GO:0005044	scavenger receptor activity	0.063360
		GO:0030544	Hsp70 protein binding	0.065914
		GO:0051219	phosphoprotein binding	0.118039
		GO:0015297	antiporter activity	0.132412
	Grey	GO:0000149	SNARE binding	0.005964
		GO:0005221	intracellular cyclic nucleotide activated monoatomic	0.011915

	cation channel activity	
GO:0043855	cyclic nucleotide-gated monoatomic ion channel activity	0.011915
GO:0099528	G protein-coupled neurotransmitter receptor activity	0.011915
GO:0045028	G protein-coupled purinergic nucleotide receptor activity	0.014067
GO:0015926	glucosidase activity	0.015141
GO:0019992	diacylglycerol binding	0.015141
GO:0016812	hydrolase activity, acting on carbon-nitrogen (but not peptide) bonds, in cyclic amides	0.016214
GO:0005248	voltage-gated sodium channel activity	0.020496
GO:0030552	cAMP binding	0.020496
GO:0001614	purinergic nucleotide receptor activity	0.021563
GO:0016502	nucleotide receptor activity	0.021563
GO:0017075	syntaxin-1 binding	0.021563
GO:0015464	acetylcholine receptor activity	0.022630
GO:0004993	G protein-coupled serotonin receptor activity	0.023695
GO:0005085	guanyl-nucleotide exchange factor activity	0.024956
GO:0016702	oxidoreductase activity, acting on single donors with incorporation of molecular oxygen, incorporation of two atoms of oxygen	0.025822
GO:0016701	oxidoreductase activity, acting on single donors with incorporation of molecular oxygen	0.026884
GO:0016411	acylglycerol O-acyltransferase activity	0.033234
GO:0020037	heme binding	0.139120

	Turquoise	GO:0005201	extracellular matrix structural constituent	0.000000
		GO:0005539	glycosaminoglycan binding	0.000000
		GO:0008017	microtubule binding	0.000000
		GO:0017116	single-stranded DNA helicase activity	0.000002
		GO:0003777	microtubule motor activity	0.000005
		GO:0003688	DNA replication origin binding	0.000054
		GO:0099507	ligand-gated monoatomic ion channel activity involved in regulation of presynaptic membrane potential	0.000075
		GO:0005518	collagen binding	0.000082
		GO:0005178	integrin binding	0.000301
		GO:0017147	Wnt-protein binding	0.001737
		GO:0008376	acetylgalactosaminyltransferase activity	0.002294
		GO:0016538	cyclin-dependent protein serine/threonine kinase regulator activity	0.002485
		GO:0008046	axon guidance receptor activity	0.002956
		GO:0004713	protein tyrosine kinase activity	0.004872
		GO:0030547	signaling receptor inhibitor activity	0.005852
		GO:0003697	single-stranded DNA binding	0.005885
		GO:0071837	HMG box domain binding	0.006130
		GO:0004222	metalloendopeptidase activity	0.006904
		GO:0045499	chemorepellent activity	0.007037
		GO:0016918	retinal binding	0.014729
		GO:0031406	carboxylic acid binding	0.023961

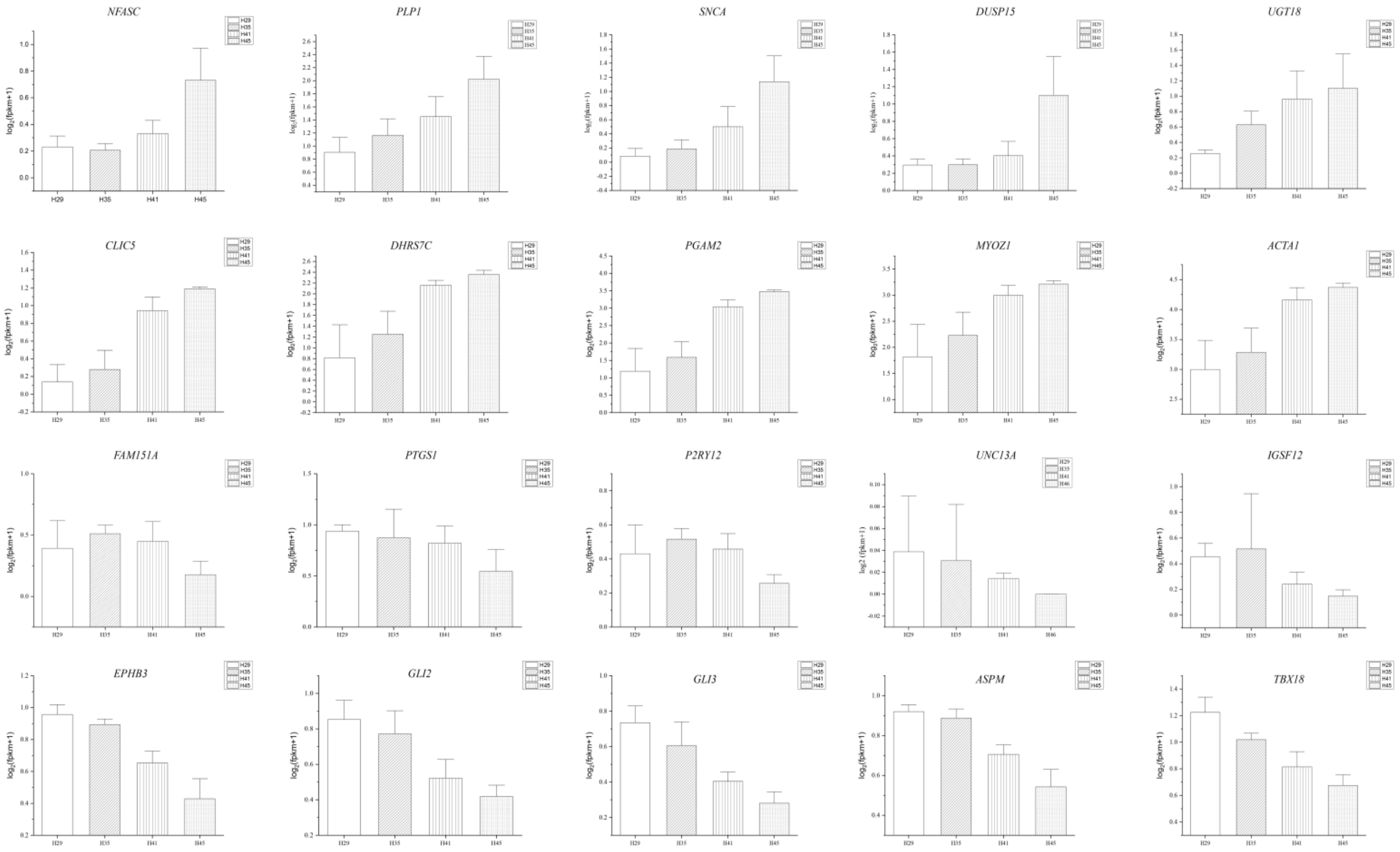

Figure S1. The expression of each hub gene in 4 developmental stages.
